# Comparative transcriptome analysis in Arabidopsis *ein2*/*ore3* and *ahk3*/*ore12* mutants during dark-induced leaf senescence

**DOI:** 10.1093/jxb/ery137

**Published:** 2018-04-10

**Authors:** Jeongsik Kim, Su Jin Park, Il Hwan Lee, Hyosub Chu, Christopher A Penfold, Jin Hee Kim, Vicky Buchanan-Wollaston, Hong Gil Nam, Hye Ryun Woo, Pyung Ok Lim

**Affiliations:** 1 Center for Plant Aging Research, Institute for Basic Science (IBS), Daegu, Republic of Korea; 2 School of Interdisciplinary Bioscience and Bioengineering, POSTECH, Pohang, Gyeongbuk, Republic of Korea; 3 Wellcome Trust/Cancer Research UK Gurdon Institute, University of Cambridge, Cambridge, UK; 4 Warwick Systems Biology Centre, University of Warwick, Coventry, UK; 5 Department of New Biology, DGIST, Daegu, Republic of Korea

**Keywords:** AHK3/ORE12, Arabidopsis, cytokinin, EIN2/ORE3, ethylene, microarray, senescence, transcriptomics

## Abstract

Leaf senescence involves degenerative but active biological processes that require balanced regulation of pro- and anti-senescing activities. Ethylene and cytokinin are major antagonistic regulatory hormones that control the timing and progression rate of leaf senescence. To identify the roles of these hormones in the regulation of leaf senescence in Arabidopsis, global gene expression profiles in detached leaves of the wild type, an ethylene-insensitive mutant (*ein2*/*ore3*), and a constitutive cytokinin response mutant (*ahk3/ore12*) were investigated during dark-induced leaf senescence. Comparative transcriptome analyses revealed that genes involved in oxidative or salt stress response were preferentially altered in the *ein2*/*ore3* mutant, whereas genes involved in ribosome biogenesis were affected in the *ahk3/ore12* mutant during dark-induced leaf senescence. Similar results were also obtained for developmental senescence. Through extensive molecular and physiological analyses in *ein2*/*ore3* and *ahk3/ore12* during dark-induced leaf senescence, together with responses when treated with cytokinin and ethylene inhibitor, we conclude that ethylene acts as a senescence-promoting factor via the transcriptional regulation of stress-related responses, whereas cytokinin acts as an anti-senescing agent by maintaining cellular activities and preserving the translational machinery. These findings provide new insights into how plants utilize two antagonistic hormones, ethylene and cytokinin, to regulate the molecular programming of leaf senescence.

## Teaser

Ethylene and cytokinin play antagonistic roles in the regulation of leaf senescence via EIN2/ORE3-dependent transcriptional regulation of stress responses and AHK3/ORE12-dependent transcriptional maintenance of the translational machinery, respectively.

## Introduction

Senescence in higher organisms is the final stage of development and occurs at the cellular, tissue, organ, and organismal level. Leaf senescence is an organ-level senescence that involves highly regulated reprogramming of gene expression and metabolism, which is required for an orderly breakdown of organelles and the remobilization of nutrients. This process thus constitutes an important phase in a plant’s life cycle contributing to its survival and sustainability (Lim *et al.*, 2007).

The onset and progression of leaf senescence must be under tight control, as premature leaf senescence can result in insufficient accumulation of photosynthetic materials, whereas delayed leaf senescence can cause inefficient utilization of nutrients needed for seed set and development of new organs. Although leaf senescence is developmentally controlled, the initiation and progression of leaf senescence are also affected by internal and external signals (Buchanan-Wollaston *et al.*, 2003; Yoshida, 2003; Lim *et al.*, 2007). For example, under stress conditions, plants employ premature leaf senescence as a defense mechanism, which induces early seed set.

Extensive reprogramming of gene expression is required for the drastic transition in metabolic processes during leaf senescence. Analyses of global gene expression profiles using microarray and RNA sequencing (RNA-Seq) approaches have been successful in elucidating the molecular mechanisms underlying leaf senescence (Lin and Wu, 2004; Buchanan-Wollaston *et al.*, 2005; van der Graaff *et al.*, 2006; Breeze *et al.*, 2011; Woo *et al.*, 2016). In agreement with the physiological changes during this process, genes related to macromolecule degradation, metabolite mobilization, and chlorophyll biosynthesis show temporal regulation. Expression of regulatory genes encoding transcription factors (TFs) and those involved in protein degradation, phosphorylation, and dephosphorylation also undergoes substantial changes. Additionally, genes involved in the biosynthesis, degradation, inactivation, and signaling pathways of hormones, such as ethylene, cytokinin, jasmonic acid (JA), salicylic acid (SA), auxin, abscisic acid, gibberellin, and brassinosteroid, are differentially expressed during leaf senescence.

Ethylene and cytokinin are key regulators of leaf senescence. Exogenous application of ethylene induces premature leaf senescence in *Arabidopsis thaliana* and tobacco (*Nicotiana tabaccum*) (Aharoni and Lieberman, 1979; Bleecker *et al.*, 1988). Direct involvement of ethylene in promoting leaf senescence was initially demonstrated using three ethylene-insensitive mutants, *ethylene resistant 1* (*etr1*), *ethylene insensitive 2* (*ein2*), and *ein3*, which exhibited greater leaf longevity than the wild type (Grbic and Bleecker, 1995). Additionally, forward genetic screening of loci controlling leaf senescence has shown that the *oresara3* (*ore3*) mutant is allelic to *ein2-1* (Oh *et al.*, 1997). The role of ethylene in leaf senescence has been further elucidated through the identification of a trifurcate feed-forward loop for age-dependent cell death involving *EIN2/ORE3*, *ORE1*, and *miR164* (Kim *et al.*, 2009). EIN3 is a TF that plays a key role in the ethylene signaling pathway downstream of EIN2 and promotes chlorophyll degradation by activating chlorophyll catabolic genes (Qiu *et al.*, 2015). Cytokinin is a major senescence-delaying plant hormone whose effects are particularly conspicuous during dark-induced senescence. Cytokinin levels decrease during leaf senescence, and exogenous application or overproduction of cytokinin delays senescence (Gan and Amasino, 1995; McCabe *et al.*, 2001). Genetic evidence for cytokinin involvement in leaf senescence has been provided by the analysis of Arabidopsis *LONELY GUY* genes (*AtLOG* genes), which encode cytokinin-activating enzymes. Ectopic overexpression of *AtLOG* genes slows the decline in chlorophyll content of leaves during dark-induced senescence (Kuroha *et al.*, 2009). The effect of cytokinin on leaf senescence is mediated by one of the cytokinin receptors, *Arabidopsis Histidine Kinase 3* (*AHK3*), whose gain-of-function mutant, *ore12-1*, was identified as a delayed leaf senescence mutant (Kim *et al.*, 2006).

In parallel with these molecular genetic studies, transcriptome analyses of genetic mutants have been performed to dissect the role of hormone signaling pathways during leaf senescence (Kim *et al.*, 2006; Yu *et al.*, 2016). However, little is known about the mechanisms by which a plant co-ordinates different hormonal signaling pathways to regulate leaf senescence. Since leaf senescence is controlled by a complex crosstalk of hormone signaling pathways, the identification of key senescence-specific pathways mediated by hormone signaling factors, such as EIN2/ORE3 and AHK3/ORE12, is very difficult. Moreover, the altered gene expression in mutants defective for senescence regulators can be largely attributed to the effect of the delayed or early senescence phenotype, which might lead to misleading conclusions.

In this study, we focused on addressing the regulatory mechanisms governing ethylene and cytokinin signaling pathways by comparing the time-course profiles of global gene expression during dark-induced leaf senescence in the wild type, the ethylene-insensitive mutant *ein2*/*ore3*, and the constitutive cytokinin response mutant *ahk3*/*ore12*. The results showed that while EIN2/ORE3 and AHK3/ORE12 share common target genes that modulate leaf senescence, they also mediate stress response and cellular maintenance processes, respectively, by regulating distinct sets of targets. We also propose distinct roles for EIN2/ORE3 and AHK3/ORE12: the former is involved in transcriptional regulation of stress responses, whereas the latter is involved in the transcriptional maintenance of the translational machinery. Collectively, this study suggests a molecular framework for antagonistic regulation of leaf senescence by EIN2/ORE3 and AHK3/ORE12, two key regulators of ethylene and cytokinin signaling, respectively.

## Materials and methods

### Plant material and growth conditions

*Arabidopsis thaliana* ecotype Columbia-0 (Col; wild type) was used in this study. The *ein2/ore3* and *ahk3/ore12* mutants have been previously described as *ein2-44*/*ore3-1* and *ore12-1*, respectively (Oh *et al.*, 1997; Kim *et al.*, 2006). Plants were grown in a growth room (Korea Instrument, Korea) under a long-day photoperiod (16 h light/8 h dark cycle) and 22 °C temperature.

### Leaf senescence assays

The third and fourth leaves of 3-week-old plants were harvested and floated on 3 mM MES (pH 5.7). The samples were incubated at 22 °C in the dark for up to 11 d. The photochemical efficiency of PSII (Lee *et al.*, 2016) and chlorophyll content (Vernon, 1960) of leaves were measured as described previously. For chemical- or plant hormone-mediated senescence assay, detached leaves were floated abaxial side up in 3 mM MES (pH 5.7) with or without 50 μM 1-aminocyclopropane-1-carboxylic acid (ACC; Sigma, USA), 0.2 μM 6-benzylaminopurine (BA; Sigma, USA), 100 mM sodium chloride (Samchun, Korea), or 10 mM hydrogen peroxide (Junsei, Japan). All treatments were performed at 22 °C in the dark. Photochemical efficiency and chlorophyll content were measured as described above. For developmental leaf senescence assays, the third and fourth rosette leaves at the indicated leaf age were harvested from individual plants and used for measurements of chlorophyll content, photochemical efficiency, and gene expression. Leaves used for gene expression and microarray experiments were harvested 4 h into the subjective day.

### Gene expression analysis

Total RNA was isolated from leaves of Col, *ein2*/*ore3*, and *ahk3*/*ore12* plants using WelPrep™ Total RNA Isolation Reagent (Welgene, Korea) and used for cDNA synthesis employing the ImProm II™ Reverse Transcriptase System kit (Promega, USA). Quantitative real-time PCR (qPCR) was performed using SYBR Premix Ex-*Taq* (Enzynomics, Korea) and the CFX96 Touch Real-Time PCR Detection System (Bio-Rad, USA). Fold changes in gene expression were calculated using the C_T_ method relative to their initial expression in leaves of each genotype at day 0 (before dark incubation) in dark-induced leaf senescence or 12 days after leaf emergence (DAE) in developmental leaf senescence and normalized against *Actin-2* (*ACT2*). Primers used for qPCRs are listed in Supplementary Table S1 at *JXB* online.

### Microarray experiments

Microarray experiments were carried out in two biological replicates using the third and fourth leaves of Col, *ein2*/*ore3*, and *ahk3*/*ore12* incubated in the dark for 0–3 d. Total RNA was isolated using a Welprep™ Total RNA Isolation Reagent (Welgene, Korea). A 10 µg aliquot of total RNA was used for cDNA synthesis using the SuperScript double-stranded cDNA synthesis kit (Invitrogen), followed by Cy3 labeling and hybridization according to NimbleGen protocols. Arrays (Athal_TAIR9_exp_H12; NimbleGen Gene Expression 12 × 135K platform) were scanned using the NimbleGen MS 200 Microarray Scanner. Array data export and analysis were performed using NimbleScan v2.5 based on the RMA algorithm. Log_2_ intensities of probes were normalized using the quantile method. Data sets are available in the NCBI GEO database (GSE99754).

### Identification of differentially expressed genes (DEGs), gene ontologies (GOs), and network densities

The expression level of a gene was calculated by adding the expression levels of all transcript variants obtained from the time-course microarrays. Expression data were imported into and analyzed using BRB ArrayTools (version 4.3.2 and R version 3.0.1; http://linus.nci.nih.gov/BRB-ArrayTools.html). Genes that were differentially expressed over time in Col were selected by ANOVA [false discovery rate (FDR) <0.05] using the ‘Time course analysis’ plug-in. Temporal gene expression profiles in Col were clustered using Short Time-series Expression Miner (STEM) software, based on the STEM clustering algorithm (Ernst and Bar-Joseph, 2006). The genes that were differentially expressed in *ein2*/*ore3* or *ahk3*/*ore12* leaves relative to Col leaves were determined using two-way ANOVA (FDR <0.05) using the ‘ANOVA for Fixed Effects Model’ plug-in, with genotype (Col versus *ein2*/*ore3* or Col versus *ahk3*/*ore12*) and time (0, 1, 2, and 3 d) as factors of variation.

GO annotation of DEGs was performed using the BiNGO 3.03 (Maere *et al.*, 2005) plug-in tool in Cytoscape version 3.22 with GO_Biological_Process, GO_Molecular_Function, and GO_Cellular_Component categories. Over-represented GO categories were identified using a hypergeometric test and Benjamini and Hochberg FDR correction (*P*<0.05) (Benjamini and Hochberg, 1995). The enrichment scores were displayed as –log_10_(*P*). The function and localization of genes involved in processes such as ‘ribosomal proteins’ and ‘protein synthesis/ribosome biogenesis’ were obtained from MAPMAN mapping files (Ath_AGI_LOCUS_TAIR10_Aug2012).

A TF co-expression network was generated using 184 differentially expressed TFs (DE-TFs) during dark-induced leaf senescence, based on Pearson’s correlation coefficient (Pcc; cut-off ≥0.99) for gene pairs of DE-TFs over time. The TF list was obtained from plant Transcription Factor Database V3.0 (Jin *et al.*, 2014). Network density was determined from the ratio of the observed number of edges to all possible edges within the network node.

## Results

### Experimental system

Although ethylene and cytokinin have long been known to induce and delay senescence, respectively, complicated crosstalk between these two hormones has made it difficult to elucidate how their antagonistic functions co-ordinate leaf senescence. In an effort to unravel the role of these two hormones in the regulation of leaf senescence, we utilized ethylene and cytokinin signaling mutants of Arabidopsis, *ein2*/*ore3* and *ahk3*/*ore12*, respectively. Both mutants show a delayed leaf senescence phenotype; *ore3*, which is allelic to *ein2*, is an ethylene-insensitive mutant, while *ore12* is a gain-of-function mutant of *AHK3* with a constitutive cytokinin response. We adopted dark-induced senescence in detached leaves as an assay to investigate changes in the physiology and global gene expression profiles of *ein2*/*ore3* and *ahk3*/*ore12* mutants during leaf senescence, as dark incubation of leaves is known to induce rapid and synchronous senescence (Liebsch and Keech, 2016).

### Effects of ethylene and cytokinin on dark-induced leaf senescence in *ein2/ore3* and *ahk3/ore12*

**Fig. 1.**
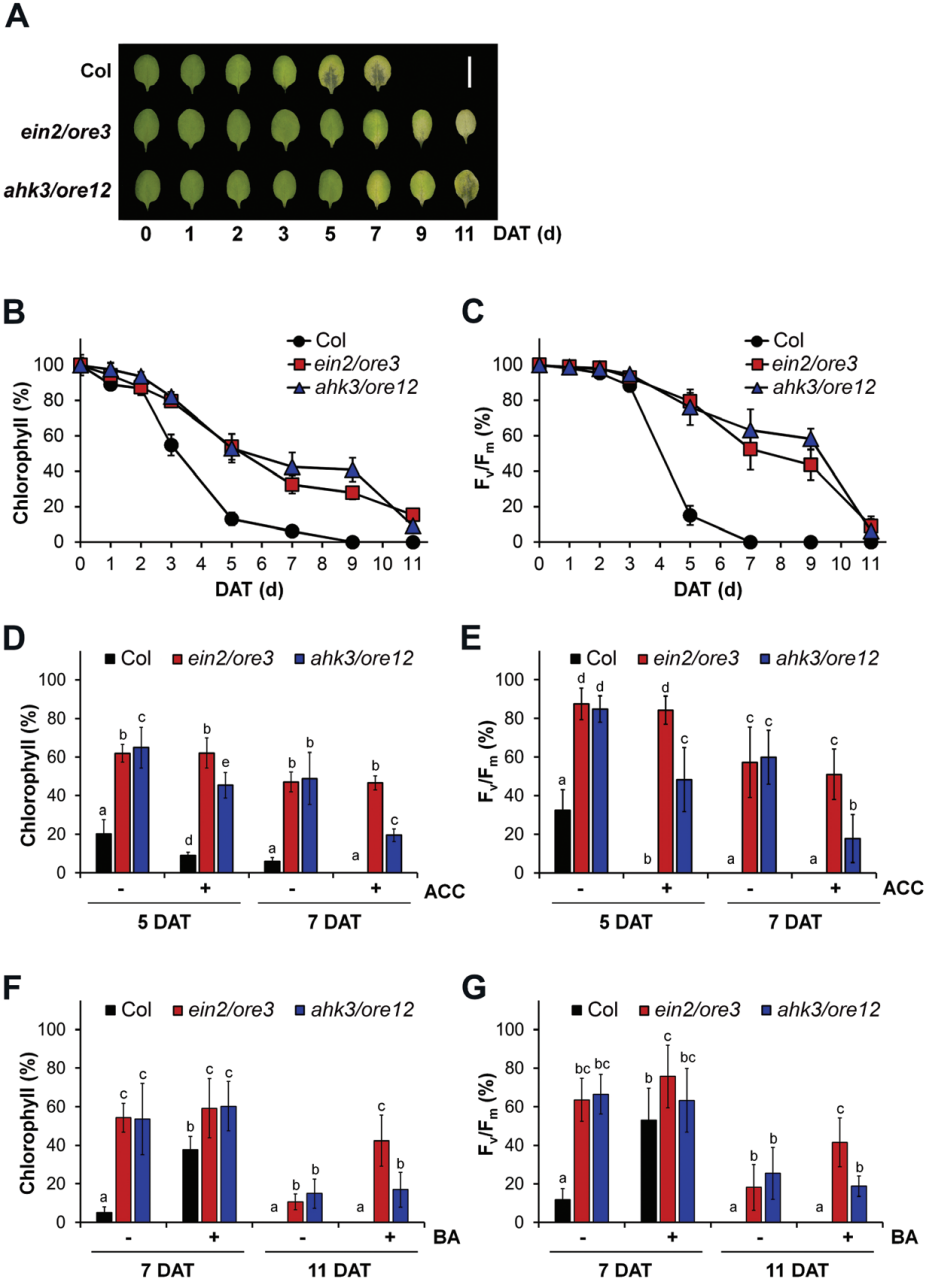
Effects of ethylene and cytokinin on dark-induced leaf senescence in Col, *ein2*/*ore3*, and *ahk3*/*ore12.* (A) Representative images, (B) chlorophyll content, and (C) photochemical efficiency of PSII (*F*_v_/*F*_m_) of Col, *ein2*/*ore3*, and *ahk3*/*ore12* leaves incubated in the dark for the specified number of days (d; DAT). Scale bar=1 cm. Data represent the mean ±SE (*n*=12). (D–G) Chlorophyll content (D, F) and photochemical efficiency of PSII (*F*_v_/*F*_m_; E, G) of leaves treated with 50 μM ACC (D, E) or 0.2 μM BA (F, G) in the dark for the specified number of days. ACC, 1-aminocyclopropane-1-carboxylic acid; BA, 6-benzylaminopurine; –/+, mock and hormone treatment. Data represent the mean ±SE (*n*=6). Statistical analyses were performed using one-way ANOVA, followed by Duncan post-hoc tests for multiple comparisons. Means with different letters above the bars indicate statistically significant results (*P*<0.05). Similar results were obtained in three independent trials.

As a first step toward comparison of senescence responses controlled by EIN2/ORE3 and AHK3/ORE12, dark-induced senescence phenotypes were monitored in leaves of Col, *ein2*/*ore3*, and *ahk3*/*ore12* plants. Third and fourth rosette leaves were detached from 3-week-old plants and incubated in the dark for up to 11 d. Leaf chlorophyll content and *F*_v_/*F*_m_-based photochemical efficiency of PSII were measured at the indicated times to monitor the progression of senescence. As a result of dark treatment, Col leaves started changing color and became pale green by 3 days after treatment (DAT), and completely yellow and/or transparent by 5 DAT, whereas *ein2*/*ore3* and *ahk3*/*ore12* leaves remained intact up to 9 DAT (Fig. 1A). Similarly, the chlorophyll content of Col leaves declined rapidly to 54.9% by 3 DAT and to 6.1% by 5 DAT of the initial chlorophyll level. In contrast, *ein2*/*ore3* and *ahk3*/*ore12* leaves retained 32.5% and 42.4% of their initial chlorophyll content at 7 DAT, respectively (Fig. 1B). The decline in the *F*_v_/*F*_m_ ratios of leaves showed a similar trend to that exhibited by the chlorophyll content (Fig. 1C). These results show that delayed senescence phenotypes of *ein2*/*ore3* and *ahk3*/*ore12* are comparable at the physiological level.

To verify that delayed leaf senescence phenotypes of *ein2*/*ore3* and *ahk3*/*ore12* mutants were the result of aberrant ethylene and cytokinin signaling, respectively, the effect of hormone application was evaluated in the dark. Leaves were treated with ACC (an immediate biosynthetic precursor of ethylene) and BA (a synthetic cytokinin) (Fig. 1D–G). Exogenous treatment of leaves with ACC in the dark accelerated the decline in the chlorophyll content and *F*_v_/*F*_m_ ratio in Col and *ahk3*/*ore12*, in comparison with the dark-only condition. However, the chlorophyll content and *F*_v_/*F*_m_ ratio of dark-incubated *ein2*/*ore3* leaves treated with ACC were comparable with those without ACC treatment (Fig. 1D, E). On the other hand, exogenous BA treatment effectively antagonized the reduction in chlorophyll content and the *F*_v_/*F*_m_ ratio by dark incubation in *ein2*/*ore3* as well as Col leaves, but failed to antagonize their reduction in *ahk3*/*ore12* leaves (Fig. 1F, G). These results indicate that delayed senescence responses in *ein2*/*ore3* and *ahk3*/*ore12* are mainly due to ethylene- and cytokinin-mediated signaling, respectively.

### Temporal profiling of transcripts during dark-induced leaf senescence

**Fig. 2.**
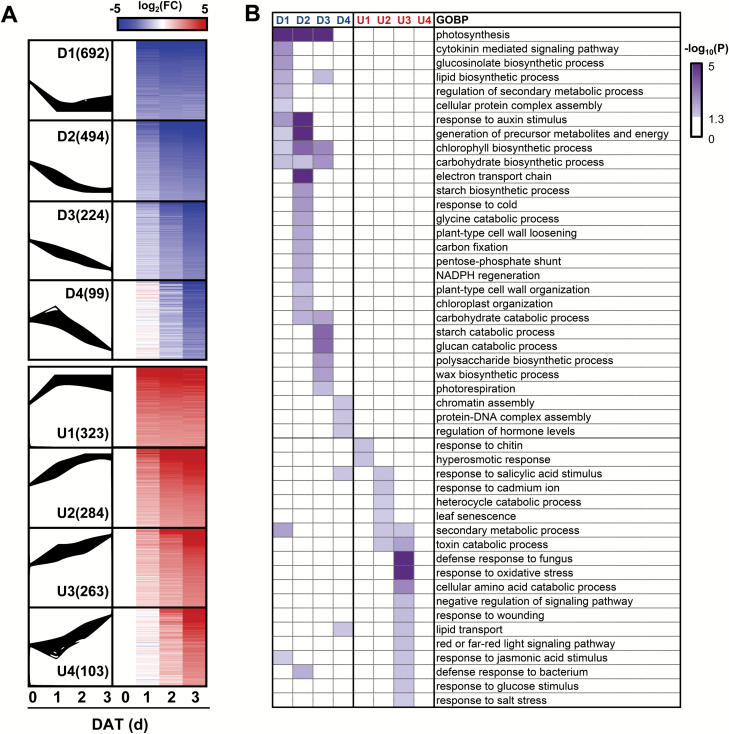
Clustering and functional classification of differentially expressed genes (DEGs) in Col during dark-induced leaf senescence. (A) Short time-series expression miner (STEM) clustering of DEGs in Col during dark-induced leaf senescence. Clusters D1–D4 comprise down-regulated genes (blue), whereas clusters U1–U4 comprise up-regulated genes (red). The number of genes in each cluster is indicated in parentheses. Auto-scaled log_2_(FC) values of gene expression in each cluster are shown in the box, and changes in gene expression relative to day 0 are shown as a heat map. FC, fold change. (B) Gene ontology biological processes (GOBPs) identified in the eight major clusters. The *P*-value is the significance of the GOBPs enriched by the DEGs in each cluster.

To understand how plants co-ordinate ethylene and cytokinin signaling pathways to regulate leaf senescence, kinetic transcriptome profiling during leaf senescence is crucial. Therefore, a time-course microarray analysis was conducted using Col, *ein2*/*ore3*, and *ahk3*/*ore12* leaves incubated in darkness for 0, 1, 2, and 3 d. A total of 2684 DEGs, corresponding to 8.2% of the total genes analyzed, were identified during dark-induced senescence (FDR <0.01, time-series analysis; see Supplementary Dataset S1). From this dataset, we first assessed the molecular basis of functional changes associated with dark-induced leaf senescence in Col. These DEGs were categorized into eight major temporal expression clusters, of which four were comprised of genes that were down-regulated (D1–D4), and four were comprised of genes that were up-regulated (U1–U4) over time in response to dark (Fig. 2A). These eight main clusters with a significantly enriched pattern (*P*<0.05) comprised 2482 genes which accounted for ~92% of DEGs. Although physiological changes of dark-incubated Col leaves were more pronounced at later time points (Fig. 1B, C), most of the DEGs started to show their expression change at 1 DAT (Fig. 2A). Only a small fraction of genes showed late responsiveness (D4, 3.7%; U4, 3.8%). These results indicate the relevance of transcriptional regulation during the early stage of dark-induced senescence.

Next, the gene ontology biological processes (GOBPs) represented by the genes in these eight clusters were identified using BiNGO software. Overall, processes involved in biogenesis and maintenance of cellular machinery were predominant among the down-regulated clusters, whereas those involved in response to external stimuli and stress-related hormones were enriched in the up-regulated clusters (Fig. 2B). In the D1 cluster, cellular assembly processes (biosynthetic process of lipid and protein complex) were mainly affected. Hormone-related processes such as the cytokinin-mediated signaling pathway and response to auxin signaling were another group of important biological processes enriched in DEGs in the D1 and/or D2 clusters. In the D2 and D3 clusters, energy metabolism (generation of precursor metabolites and energy, electron transport chain, starch biosynthesis, carbon fixation, and photorespiration) and cellular maintenance processes (cell wall and chloroplast organization) were preferentially affected. The late down-regulated cluster (D4) includes chromatin assembly processes. On the other hand, responses to the external stimuli and stress-related hormones were mostly up-regulated during dark-induced senescence (Fig. 2B). For example, responses to chitin and cadmium were found in the U1 and U2 clusters, respectively, and responses to oxidative stress, wounding, pathogen, glucose, and salt were greatly enriched in the U3 cluster. GOBPs of response to SA and JA were enriched in U2 and U3 clusters, respectively. In addition, catabolic processes (heterocycle, toxin, and amino acid catabolic processes) were found as another major GOBP associated with genes in the U2 and U3 clusters. Collectively, dark-induced leaf senescence accompanies massive transcriptional changes of the genes involved in a variety of functional processes that would be temporally co-ordinated.

### Comparative transcriptome analysis in *ein2/ore3* and *ahk3/ore12* during dark-induced leaf senescence

**Fig. 3.**
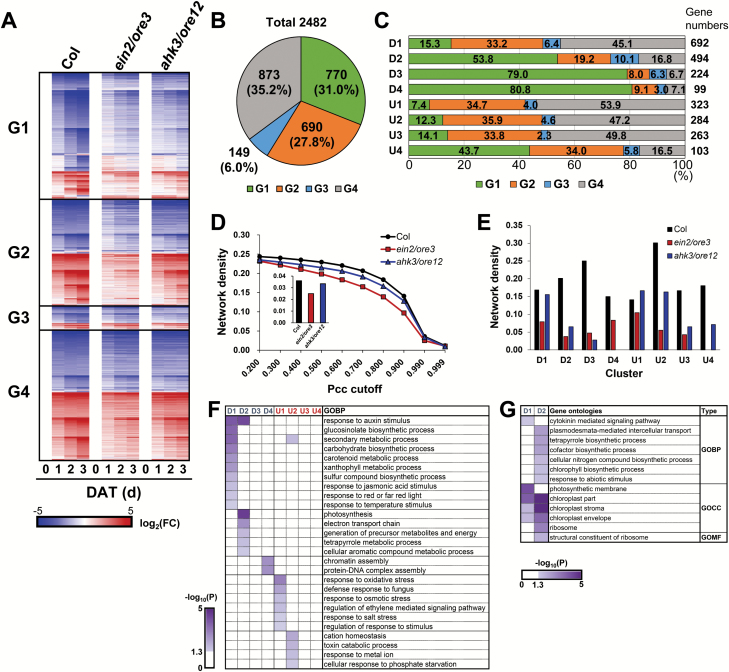
Comparative analysis of genes differentially expressed in the *ein2*/*ore3* and *ahk3*/*ore12* transcriptome during dark-induced leaf senescence. (A) Relative expression of genes belonging to four response groups: G1, affected in both *ein2*/*ore3* and *ahk3*/*ore12*; G2 and G3, affected in *ein2*/*ore3* and *ahk3*/*ore12*, respectively; and G4, not affected in either *ein2*/*ore3* or *ahk3*/*ore12*. DEGs in each mutant were identified using two-way ANOVA (FDR <0.05). Gene expression is shown as a heat map as described in Fig. 2A. (B) Proportion of genes in each group. (C) Proportion of genes in each cluster. (D) Network density of the transcription factor (TF) co-expression network as a function of the Pearson’s correlation coefficient (Pcc) cut-off value in Col, *ein2*/*ore3*, and *ahk3*/*ore12.* Edge was defined as a link between two nodes with a Pcc greater than the cut-off value. The number of nodes was counted when connected by at least one edge. TFs in the gene list described in Fig. 2A were used. (E) Network density of the TF co-expression network containing nodes within each expression cluster in Col, *ein2*/*ore3*, and *ahk3*/*ore12*. (F, G) GOs represented by DEGs belonging to each cluster in G2 (F) and G3 (G). Color bars represent an enrichment of gene ontologies from DEGs in each cluster, shown as –log_10_(*P*-values).

To elucidate the role of ethylene and cytokinin signaling in dark-induced leaf senescence, comparative transcriptome analyses of *ein2*/*ore3* and *ahk3*/*ore12* mutants was performed. Of the 2482 DEGs in the eight main clusters, genes with altered expression in *ein2*/*ore3* and *ahk3*/*ore12* leaves were determined using two-way ANOVA (FDR <0.05). These genes were categorized into four groups: G1 (770), genes affected in both *ein2*/*ore3* and *ahk3*/*ore12* mutants; G2 (690) and G3 (149), genes affected in *ein2*/*ore3* and *ahk3*/*ore12* mutants, respectively; and G4 (873), genes with no significant change in either mutant (Fig. 3A, B). Approximately half of the genes differentially expressed in either *ein2*/*ore3* or *ahk3*/*ore12* were common to both mutants [47.9%, G1/(G1+G2+G3)], indicating considerable shared functions of *ein2*/*ore3* and *ahk3*/*ore12* at the transcript level during dark-induced leaf senescence. Moreover, the number of genes uniquely altered in *ein2*/*ore3* (690, G2) was 4.6-fold greater than that altered in *ahk3*/*ore12* (149, G3). Even within G1, the degree of altered expression was greater in *ein2*/*ore3* than in *ahk3*/*ore12* (Fig. 3A). These results are quite intriguing given that senescence progression was similar in both mutants (Fig. 1A–C).

To explore this dataset further, genes belonging to each group were subcategorized into the clusters D1–D4 and U1–U4 described above. This analysis revealed several interesting features of the transcript profiles of genes affected by *ein2*/*ore3* and *ahk3*/*ore12* mutations (Fig. 3C). First, a large proportion of genes that were common targets of EIN2/ORE3 and AHK3/ORE12 (G1) belonged to D3 or D4 (79–81%) and U4 (43.7%) clusters, suggesting that G1 genes largely influence late responses during dark-induced leaf senescence, relative to genes of other groups. Secondly, a greater proportion of genes in D1 and U1 clusters were represented by those altered in the *ein2*/*ore3* mutant (G1+G2; 48.5% in D1 and 42.1% in U1) than those altered in the *ahk3*/*ore12* mutant (G1+G3; 21.6% in D1 and 11.4% in U1), suggesting that EIN2/ORE3 is important in regulating early response genes at the transcriptional level during dark-induced senescence. Thirdly, the *ein2*/*ore3* (G1+G2) mutation influenced both up- and down-regulated genes, whereas *ahk3*/*ore12* (G1+G3) preferentially affected down-regulated genes, most of which were related to biogenesis and maintenance of cellular machinery. These results suggest different action mechanisms of EIN2/ORE3 and AHK3/ORE12 in the regulation of leaf senescence; EIN2/ORE3 accelerates leaf senescence by regulating the mRNA abundance of senescence-associated genes during the early stages, whereas AHK3/ORE12 delays leaf senescence by maintaining the mRNA level of genes involved in biosynthetic and homeostatic processes.

Because TFs play key roles in affecting global gene expression changes, co-expression networks involving 184 TFs of DEGs in Col, *ein2*/*ore3*, and *ahk3*/*ore12* leaves were examined. As the Pcc cut-off increased, the number of nodes and edges in the network (network density) decreased and reached saturation beyond a Pcc of 0.99 (Fig. 3D; Supplementary Fig. S1A). Network density was greatly reduced in *ein2*/*ore3* in comparison with *ahk3*/*ore12* and Col across a wide range of Pcc cut-off values (0.2–0.999) (Fig. 3D). This reduction in network density in *ein2*/*ore3* was observed in all clusters, and the decrease in network density in *ahk3*/*ore12* occurred to a lesser degree in U2–U4 clusters, in comparison to *ein2/ore3* (Fig. 3E; Supplementary Fig. S1B). Nonetheless, *ahk3*/*ore12* had a similar level of network density in D1 and U1 clusters, relative to Col. These data further support the involvement of EIN2/ORE3 in regulating gene transcription during the early stages of dark-induced senescence.

To dissect the biological processes affected by *ein2*/*ore3* and *ahk3*/*ore12* mutations during dark-induced senescence, GOs in G2 and G3 groups were compared. Genes specifically altered in *ein2*/*ore3* (G2) were associated with a wide range of processes, including epigenetic regulation, stress response, maintenance of homeostasis, and regulation of ethylene-mediated signaling (Fig. 3F). In contrast, the G3 group was associated with a relatively small number of GOBPs, including various biosynthetic processes and cytokinin-mediated signaling (Fig. 3G). Gene ontology cellular compartment (GOCC) and gene ontology molecular function (GOMF) enrichment revealed association of G3 genes with chloroplast and ribosome structure (Fig. 3G). Overall, these data indicate the preferential involvement of *EIN2*/*ORE3* in stress-related responses and that of *AHK3*/*ORE12* in chloroplast integrity or self-maintenance processes during dark-induced leaf senescence.

Expression profiles of genes grouped into biological processes that are dependent on EIN2/ORE3- or AHK3/ORE12-specific signaling pathways were further confirmed by qPCR (Supplementary Figs S2, S3). The induction of genes involved in response to salt stress (*AT4G16260*, *PR4*, and *RAP2.6*) and oxidative stress (*AT1G21520*, *AT4G08780*, *ATLEA5*, and *PRX37*) was attenuated to a greater degree in dark-treated leaves of *ein2*/*ore3* than those of *ahk3*/*ore12* (Supplementary Fig. S2A, B). Furthermore, to confirm whether altered expression of these genes in *ein2*/*ore3* was due to defects in ethylene signaling, we monitored the effect of Ag (I), an ethylene inhibitor, on transcriptional changes during dark incubation. Ag (I) treatment attenuated the induction of several stress-response genes, including *AT4G16260*, *PR4*, *AT1G21520*, and *ATLEA5* in Col and *ahk3*/*ore12*, but not in *ein2*/*ore3* (Supplementary Fig. S2C, D). Similarly, the down-regulation of genes related to cytokinin signaling (*AHK4* and *ARR4*), tetrapyrrole or chlorophyll biosynthesis (*CHLI2* and *GSA2*), and ribosome structure (*CPN60B, EMB3105*, *PRPL28*, and *RPL9*) in *ahk3*/*ore12* was less than that in *ein2*/*ore3* (Supplementary Fig. S3A–C), indicating that changes in gene expression observed in the *ahk3*/*ore12* mutant are dependent on cytokinin signaling. Moreover, BA treatment suppressed the decrease in the expression of genes associated with the cytokinin-mediated signaling pathway and ribosome complex in Col and *ein2*/*ore3*, but such an effect was not observed in *ahk3*/*ore12* (Supplementary Fig. S3D, E). Together, these results further support the involvement of ethylene and cytokinin signaling in dark-induced leaf senescence. Moreover, these data indicate that transcriptional changes of stress-responsive genes and ribosomal protein (RP) genes during dark-induced senescence are prominently due to EIN2/ORE3-mediated ethylene signaling and AHK3/ORE12-mediated cytokinin signaling, respectively.

### EIN2/ORE3 up-regulates genes involved in salt and oxidative stress response in the dark

Given that the increased expression of genes related to oxidative and/or salt stress was attenuated in *ein2*/*ore3* during dark-induced leaf senescence (Fig. 3F; Supplementary Fig. S2), we inferred that the *ein2*/*ore3* mutation might alter salt and oxidative stress responses during dark-induced leaf senescence.

**Fig. 4.**
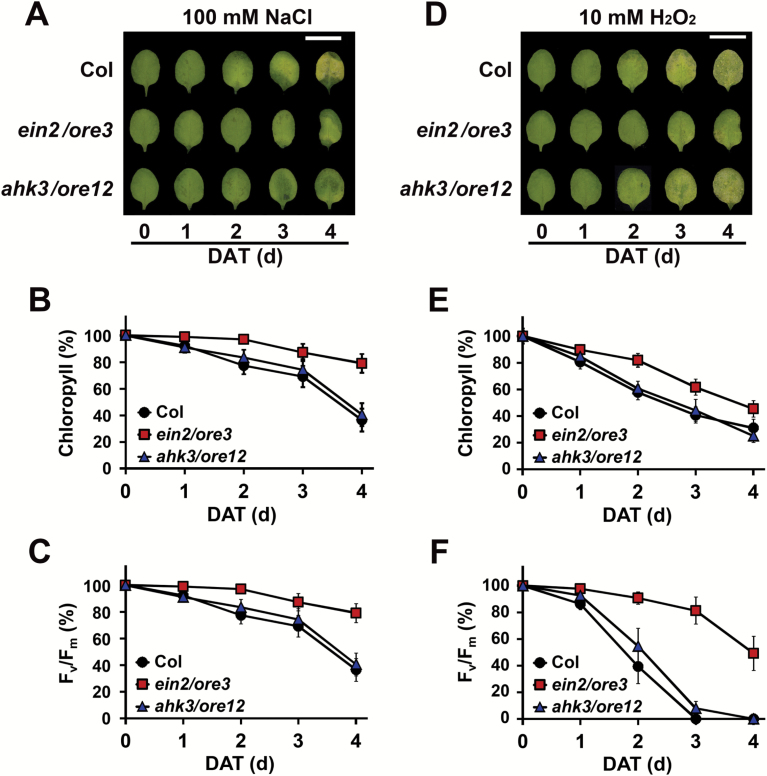
EIN2/ORE3-mediated salt and oxidative stress response during dark-induced leaf senescence. Leaves of Col, *ein2*/*ore3*, and *ahk3*/*ore12* were treated with 100 mM NaCl (A–C) or 10 mM H_2_O_2_ (D–F) in the dark for the specified number of days (d; DAT) indicated. Representative images (A, D), and measurements of chlorophyll content (B, E) and photochemical efficiency of PSII (*F*_v_/*F*_m_) (C, F) of leaves are shown. Scale bar (A, D)=1 cm. Data represent the mean ±SE (*n*=24). Similar results were obtained in three independent trials.

To obtain experimental evidence for the involvement of the *ein2*/*ore3* mutation in salt and oxidative stress responses, leaves of Col, *ein2*/*ore3*, and *ahk3*/*ore12* were treated with 100 mM NaCl or 10 mM H_2_O_2_ in the dark for 0–4 d. Disintegration of leaf tissue marked by yellowing and necrosis of leaves was observed at 3 DAT in leaves of Col and *ahk3*/*ore12* in response to both salt and oxidative stress, whereas leaves from *ein2*/*ore3* remained green and intact (Fig. 4A, D). Similarly, the chlorophyll content and *F*_v_*/F*_m_ ratio of *ein2*/*ore3* leaves were much higher than those of Col and *ahk3*/*ore12* leaves (Fig. 4B, C, E, F). These results indicate that EIN2/ORE3, at least in part, plays an important role in promoting dark-induced leaf senescence by activating salt and oxidative stress response genes and/or signaling pathways.

### Self-maintenance function of *AHK3/ORE12* during dark-induced leaf senescence

**Fig. 5.**
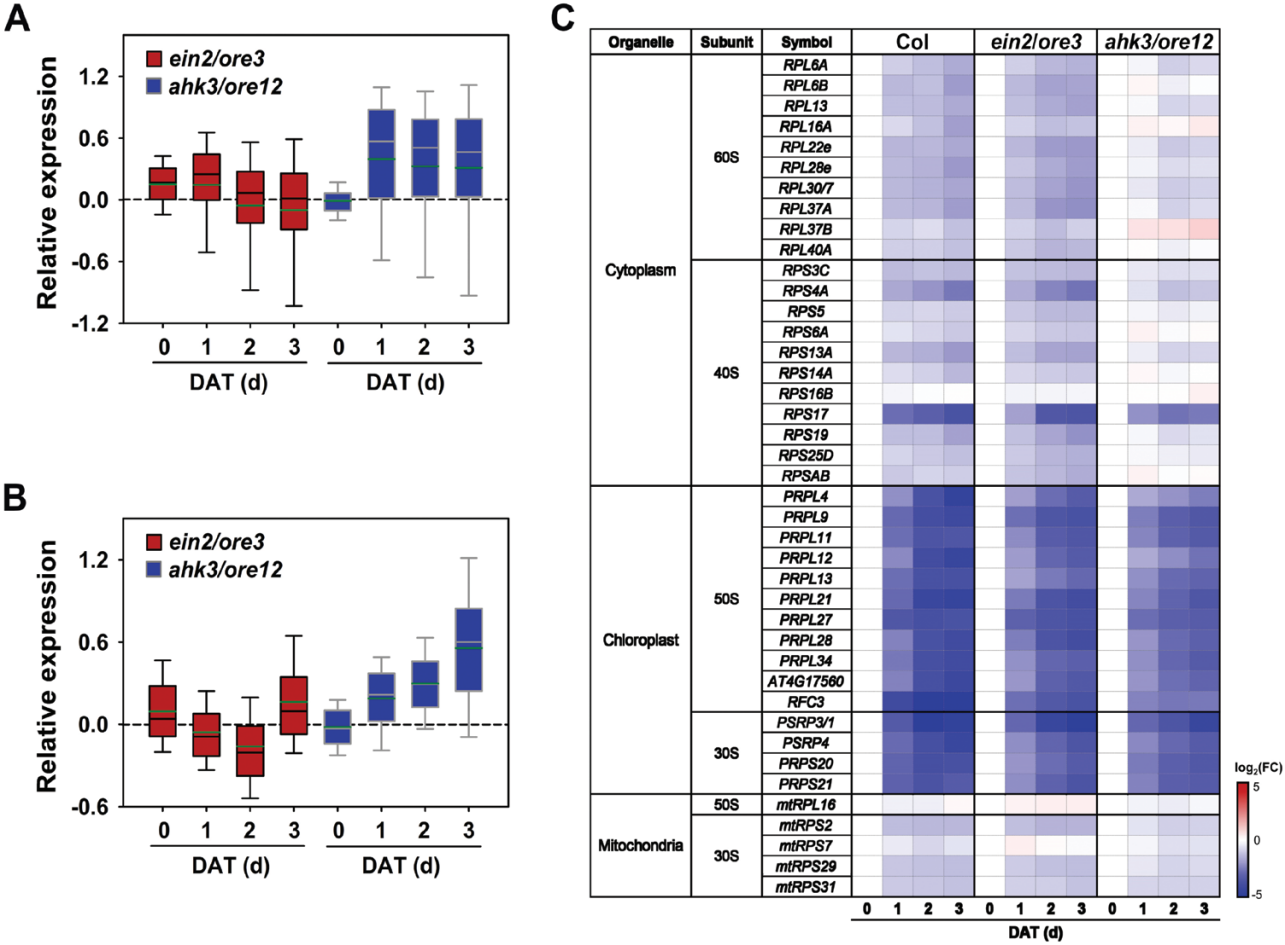
Expression of genes involved in the biogenesis of the ribosome complex in Col, *ein2*/*ore3*, and *ahk3*/*ore12* during dark-induced leaf senescence. (A, B) Box-plots showing expression of genes encoding ribosomal proteins (RPs) (A) and proteins involved in the process of ribosome biogenesis (B) in *ein2*/*ore3* and *ahk3*/*ore12* leaves relative to that in Col at the indicated number of days of dark treatment. (C) A heat map showing transcript levels of genes encoding differently localized RPs in Col, *ein2*/*ore3*, and *ahk3*/*ore12* leaves during dark-induced senescence. *ACT2* was used as the internal control. Gene expression was determined by quantitative PCR (qPCR) from three independent biological samples. Each cell shows the log_2_ ratio of gene expression at the indicated time relative to that at day 0 in each genotype.

To test the hypothesis that *AHK3*/*ORE12* is involved in the transcriptional maintenance of the translational machinery involved in self-maintenance processes, such as ribosome biogenesis during dark-induced senescence, we analyzed expression profiles of *RP* genes and of those involved in ribosome biogenesis-related processes including assembly factors, pre-RNA processing, modification factors, and exporting proteins. The temporal expression profile of *RP* genes in the *ein2*/*ore3* and *ahk3*/*ore12* mutants relative to that in Col was evaluated during dark incubation. As indicated in box-plots (Fig. 5A), *RP* transcripts accumulated to higher levels in *ahk3*/*ore12* mutants than in Col at 1, 2, and 3 DAT but not on 0 DAT. Conversely, the *ein2*/*ore3* mutation showed a minor effect on the expression of *RP* genes, when compared with Col (Fig. 5A). Similar data were obtained for genes involved in ribosome biogenesis in *ahk3*/*ore12* (Fig. 5B).

We further extended our analysis by examining the expression profiles of *RP* genes whose proteins are localized to the cytoplasm, chloroplast, and mitochondria, respectively, in independently isolated RNAs using qPCR. In both Col and *ein2*/*ore3* leaves, transcripts of cytoplasmic and mitochondrial *RP* genes decreased slowly over time and those of chloroplast RPs declined rapidly. However, the expression of cytoplasmic and mitochondrial *RP* genes was stable and the decline of chloroplast *RP* gene expression was attenuated in *ahk3*/*ore12* leaves, in comparison with *ein2*/*ore3* (Fig. 5C). Similar to genes involved in structural constituents of the ribosome, the expression of cytosolic and mitochondrial *RP* genes was stabilized in Col and *ein2*/*ore3*, but not in *ahk3*/*ore12*, following BA treatment (Supplementary Figs S3E, S4). Together, these results suggest that AHK3/ORE12 is involved in self-maintenance activities, such as ribosome biogenesis, by stabilizing *RP* expression during dark-induced leaf senescence.

### Roles of EIN2/ORE3 and AHK3/ORE12 in developmental leaf senescence

**Fig. 6.**
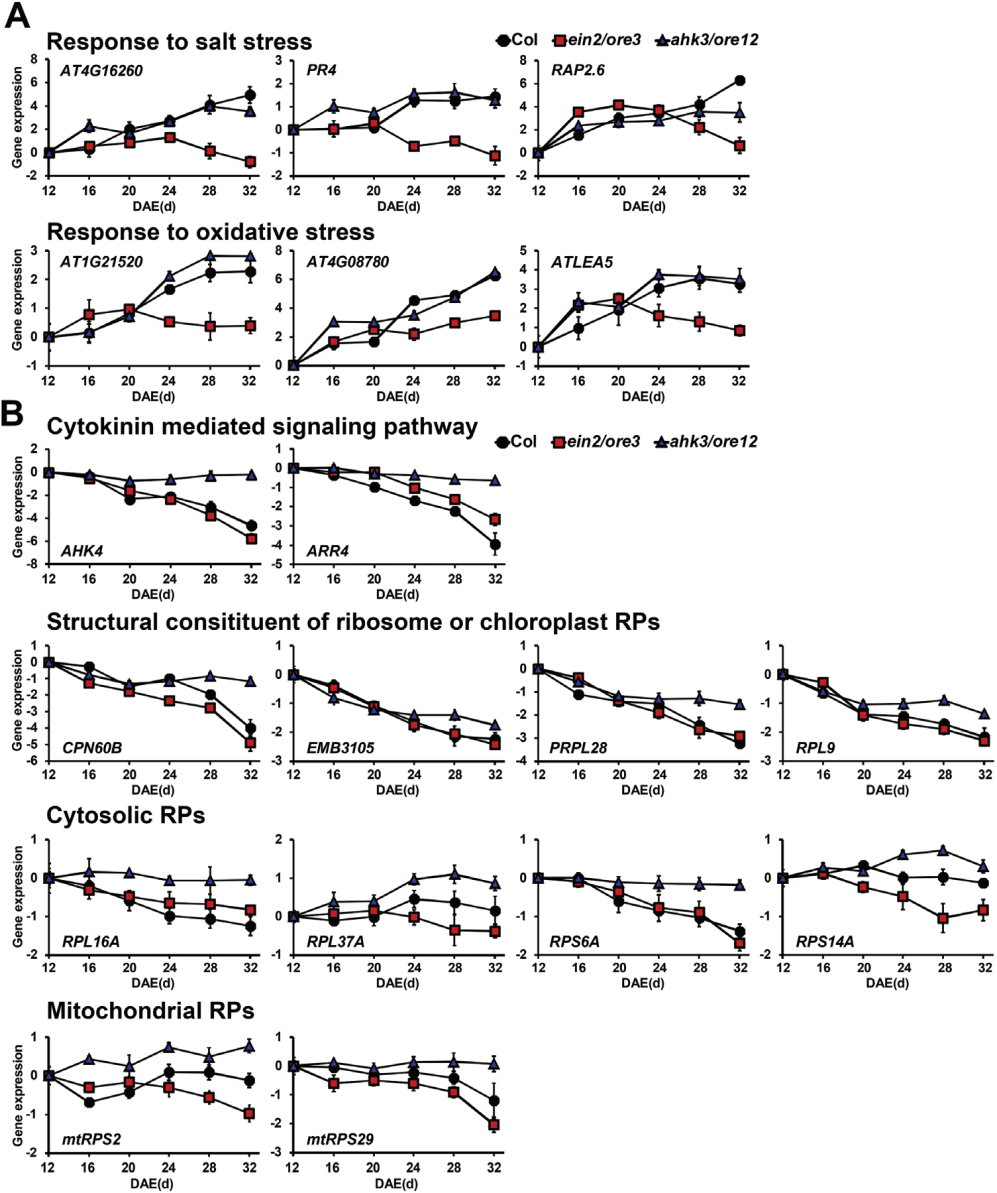
EIN2/ORE3 and AHK3/ORE12 are involved in the transcriptional regulation of stress responses and ribosome biogenesis during developmental senescence. (A, B) qPCR showing transcript levels of genes related to salt and oxidative stress response (A) and cytokinin signaling or ribosome biogenesis (B) in Col, *ein2*/*ore3*, and *ahk3*/*ore12* leaves of the indicated ages (d; DAE, days after leaf emergence) during developmental senescence. *ACT2* was used as the internal control. Kinetic patterns of gene expression are shown as the log_2_ ratio of gene expression at the indicated day (d; DAE) relative to that at 12 DAE in each genotype. Data represent the mean ±SE (*n*=4).

Dark-induced senescence, in large part, shares common execution programs with age-dependent developmental senescence (Buchanan-Wollaston *et al.*, 2005; van der Graaff *et al.*, 2006). We thus analyzed the expression of stress-response genes (altered in *ein2*/*ore3*) and *RP* genes (altered in *ahk3*/*ore12*) during developmental leaf senescence. Their transcript levels were measured every 4 d from mature (12 days after leaf emergence; DAE) to senescent (32 DAE) stages of the third and fourth leaves of Col. Expression of stress-response genes, including *AT4G16260*, *PR4*, *RAP2.6*, *AT1G21520*, *AT4G08780*, and *ATLEA5*, was increased in Col and *ahk3*/*ore12*, but was not altered in *ein2*/*ore3* during developmental leaf senescence (Fig. 6A). On the other hand, transcript levels of cytokinin signaling genes and ribosome structure-related or *RP* genes localized to the cytoplasm, chloroplast, or mitochondria accumulated to higher levels in *ahk3*/*ore12* than in Col and *ein2*/*ore3* during developmental leaf senescence (Fig. 6B). Notably, a similar progression of developmental senescence was observed in *ein2*/*ore3* and *ahk3*/*ore12* mutants when they were examined using physiological and molecular markers (Supplementary Fig. S5), indicating that differential transcriptional changes of target genes between *ein2*/*ore3* and *ahk3*/*ore12* are not due to differences in senescence progression in these mutants. Collectively, EIN2/ORE3-mediated stress gene regulation and AHK3/ORE12-mediated *RP* gene regulation are required for both dark-induced and developmental leaf senescence.

## Discussion

Leaf senescence involves hundreds of developmentally regulated processes that require a sophisticated balance between pro- and anti-senescing elements (Lim and Nam, 2005; Lim *et al.*, 2007). Ethylene and cytokinin are well-known endogenous factors that promote and delay leaf senescence, respectively (Gan and Amasino, 1995; Grbic and Bleecker, 1995; Khan *et al.*, 2014). In this study, we compared temporal transcriptome profiles during dark-induced leaf senescence in *ein2*/*ore3* and *ahk3*/*ore12* mutants, both of which show delayed senescence due to alteration of ethylene and cytokinin signaling, respectively (Fig. 1D–G) (Oh *et al.*, 1997; Kim *et al.*, 2006). Our studies revealed that stress-mediated responses and ribosomal biogenesis are key biological processes regulated by EIN2/ORE3 and AHK3/ORE12, respectively. Additionally, our data suggest that primary molecular functions of EIN2/ORE3 and AHK3/ORE12 for the regulation of target genes probably operate at different regulatory layers: transcriptional and translational layers, respectively (Figs 3, 5). Together, our findings provide a molecular basis for the co-ordinated regulation of senescence by two antagonistic hormones, ethylene and cytokinin.

### Experimental assays used in this study

To determine the molecular basis of leaf senescence, several experimental approaches were adopted in this study. Time-course analysis of global gene expression was performed to study dark-induced senescence of detached leaves. Dark-induced leaf senescence is advantageous as it results in synchronized leaf senescence regardless of the developmental stage of cells within an individual leaf, making this technique highly effective and reproducible. This might alleviate the complexity due to the lack of co-ordinated development of cells in a developing leaf. Additionally, the effect of cytokinin application on delaying leaf senescence is greater in detached leaves incubated in the dark than in intact leaves (Li *et al.*, 1992; Gan and Amasino, 1995). Arabidopsis *ein2*/*ore3* and *ahk3*/*ore12* mutants were used for the elucidation of the effects of ethylene and cytokinin on dark-induced leaf senescence, instead of treating them with cytokinin and ethylene exogenously. Comparing the transcriptome of two delayed senescence mutants with similar senescence responses under the same experimental conditions (Fig. 1A–C) helped avoid the introduction of uncontrolled variability in the experiments. Additionally, our conclusions were further strengthened by the biological and molecular consequences of cytokinin, ethylene, or ethylene inhibitor application to rule out unpredicted side functions of *ein2*/*ore3* and *ahk3*/*ore12* mutations independent of their roles in hormone signaling (Fig. 1D–G; Supplementary Figs S2C, D, S3D, E, S4). It should be noted that conclusions derived from the dark-induced senescence assay were also obtained in age-dependent developmental senescence assays (Fig. 6).

### Transcriptome profiling during dark-induced senescence

Genome-wide transcriptome analysis has been previously performed in Arabidopsis to understand the physiology of dark-induced senescence (Lin and Wu, 2004). However, the experimental assay used whole plants incubated in the dark, instead of detached leaves, as used in our study. The physiological responses of whole plants reported by Lin and Wu (2004) were later reported to be conspicuously different from those of detached leaves incubated in the dark (Weaver and Amasino, 2001).

In this study, several statistical analyses for microarray data including ANOVA-based time-series analysis, STEM-based identification of significant clusters, and GO enrichment tests (Fig. 2) were applied, allowing informative clustering and identification of DEGs and signaling pathways. Detailed expression clustering and GO enrichment categories suggested that down-regulated clusters (D1–D4) were largely associated with GOs involved in biogenesis and homeostasis, whereas up-regulated clusters (U1–U4) were mostly associated with GOs such as responses to external stimuli and stress-related hormones. In addition, cytokinin signaling and auxin response genes were enriched in the D1 cluster, while SA and JA response genes were enriched in U2 and U3 clusters, respectively. Gene expression patterns of hormone signaling and stress-response genes in dark-induced leaf senescence were similar to those in developmental leaf senescence, when compared with previous transcriptome data from other types of leaf senescence (Buchanan-Wollaston *et al.*, 2005; Breeze *et al.*, 2011; Woo *et al.*, 2016). In addition to the role of hormone signaling in leaf senescence, our results also support the involvement of other metabolic pathways such as nitrogen remobilization and starch catabolism. Differential utilization of nitrogen reserves has been observed between developmental leaf senescence and senescence of dark-adapted intact leaves (Buchanan-Wollaston *et al.*, 2005). Additionally, down-regulation of genes associated with chromatin regulation, observed for the first time in our analysis, may explain the irreversibility of dark-induced physiological changes that are not recovered following light incubation (Weaver and Amasino, 2001).

Many genes involved in biosynthetic and homeostatic processes grouped together in clusters composed of late-responsive and/or down-regulated genes during dark-induced senescence. Our results further showed that these biological processes were altered in both *ein2*/*ore3* and *ahk3*/*ore12* mutants, indicating that biosynthetic and homeostatic processes are regulated by two hormones in an antagonistic fashion. Collectively, our data provide a comprehensive overview of temporal transcript profiling, functional enrichment, and hormone signaling associated with dark-induced senescence.

### EIN2/ORE3-mediated ethylene signaling in the regulation of dark-induced senescence

Ethylene, a volatile hormone, acts as a strong inducer of systemic senescence in a variety of plant species (Abeles *et al.*, 1988; Jing *et al.*, 2005). Biosynthesis of ethylene is triggered in senescing leaves, flowers, and ripened fruits. The role of ethylene in leaf senescence has been investigated using genetic approaches, which has resulted in the identification of ethylene signaling mutants, such as *etr1* and *ein2/ore3* (Grbic and Bleecker, 1995; Oh *et al.*, 1997). EIN2 is a central molecule in ethylene signaling, and its loss-of-function mutations lead to insensitivity to ethylene (Alonso *et al.*, 1999). EIN2/ORE3-mediated senescence regulatory cascades have been partially identified; EIN2/ORE3 functions as an upstream molecule of EIN3/EIN3-LIKE 1 TF, which directly regulates *miR164* as well as *ORE1* and *AtNAP*, two key NAC TF genes governing induction of many senescence-associated NAC TF genes (Balazadeh *et al.*, 2010; Li *et al.*, 2013; Kim *et al.*, 2014). EIN2/ORE3 also mediates activation of another group of *NAC* genes such as *ANAC019*, *ANAC047*, *ANAC055*, and *ORE1 SISTER1* via EIN3-independent pathways during senescence (Kim *et al.*, 2014). Senescence-associated NAC TFs are tightly associated with stress responses (Balazadeh *et al.*, 2010). It was also found that EIN2/ORE3 is required for the expression of some of these senescence NAC TFs including ORE1, one of the master regulators of leaf senescence (Kim *et al.*, 2009).

The significance of EIN2/ORE3 in ethylene-mediated senescence is evident from our data. Transcriptome analyses revealed that expression of senescence-associated genes and of genes encoding TFs as well as the degree of co-expression of TFs were greatly affected in *ein2*/*ore3* during dark-induced senescence (Fig. 3A–E). In contrast to *ahk3*/*ore12*, *ein2*/*ore3* affected not only down-regulated DEGs, but also a significant portion of up-regulated DEGs. In the D1 cluster, JA-mediated responses were affected in *ein2*/*ore3*, which explains the previous report of an impaired JA response in the *ein2* mutant during dark-induced senescence (Li *et al.*, 2013). Our transcriptome data also showed that *EIN2*/*ORE3* regulates the ethylene-mediated signaling pathway as well as multiple stress-response processes including salt and oxidative stress enriched in the U1 cluster (Fig. 3F), which is consistent with studies reporting that ethylene is involved in the regulation of stress responses (Cao *et al.*, 2007; Asensi-Fabado *et al.*, 2012). We further confirmed that salt and oxidative stress responses in dark-induced leaf senescence were attenuated in *ein2*/*ore3* and in leaves treated with an ethylene inhibitor (Fig. 4; Supplementary Fig. S2). The requirement for EIN2/ORE3 for the expression of genes related to salt and/or oxidative stress was also observed during developmental leaf senescence (Fig. 6). Thus, it is likely that ethylene plays a role in the regulation of leaf senescence via at least the EIN2/ORE3-mediated salt and oxidative stress signaling pathways. Furthermore, data showing a larger number of senescence-associated TF genes affected and the more rapid decline of TF co-expression network density in *ein2*/*ore3* than in *ahk3*/*ore12* (Fig. 3D, E) strongly suggest that EIN2/ORE3-mediated leaf senescence regulation preferentially occurs at the transcriptional level via the TF regulatory cascade. These findings provide new insights into the global regulatory mechanism affecting co-ordinated expression of many senescence genes via EIN2/ORE3.

### AHK3/ORE12-mediated cytokinin signaling in the regulation of dark-induced senescence

Cytokinin is known to delay leaf senescence. Arabidopsis AHK3/ORE12 plays a prominent role in mediating cytokinin-dependent senescence responses, while AHK2 and AHK4 are involved in mediating many other cytokinin responses. The unique and specialized function of AHK3/ORE12 was confirmed using the constitutive cytokinin signaling mutant, *ahk3*/*ore12*. The effect of cytokinin in delaying leaf senescence is highly attenuated in the *ahk3*/*ore12* mutant, further demonstrating that senescence responses in *ahk3*/*ore12* represent cytokinin-mediated effects. This report also showed that the phosphorylation of Arabidopsis response regulator 2 (ARR2) mediated by AHK3/ORE12 is important for the regulation of gene expression in leaf longevity (Kim *et al.*, 2006). Cytokinin contents decrease at the onset of senescence, possibly through cytokinin metabolic enzymes, which constitutes another regulatory layer linking cytokinin signaling to leaf senescence control (Buchanan-Wollaston *et al.*, 2005; Breeze *et al.*, 2011; Woo *et al.*, 2016).

Cytokinin plays diverse roles in delaying senescence by potentially maintaining diverse cellular processes, such as chloroplast activity and cell division, and maintaining metabolic processes, such as nitrogen assimilation, source to sink nutrient transfer, and sugar metabolisms (reviewed in Zwack and Rashotte, 2013; Khan *et al.*, 2014). Our results provide molecular evidence for the involvement of cytokinin in these processes. For example, chlorophyll biosynthetic processes and plasmodesmata-mediated intercellular transport processes were less attenuated in *ahk3*/*ore12* (Fig. 3G), which might be related to the retention of chlorophyll accumulation and the sink to source transition.

Additionally, we explored the transcriptional regulation of ribosome and its related protein synthetic processes as another cytokinin-regulated mechanism that could contribute to self-maintenance and subsequent repression of senescence. Our transcriptome data showed that while the expression of chloroplast *RP* genes rapidly declined during dark incubation, that of cytoplasmic *RP* genes was sustained over time. Notably, chloroplast *RP* genes were one of the groups with higher expression in *ahk3*/*ore12* than in Col and even *ein2*/*ore3* (Figs 3G and 5C; Supplementary Fig. S3C, E ). Expression of the cytoplasmic and mitochondrial *RP* genes was also maintained at higher levels in *ahk3*/*ore12* during dark incubation, although no noticeable difference was observed before dark incubation (Fig. 5A–C; Supplementary Fig. S4). Transcriptional regulation of *RP* genes in *ahk3*/*ore12* was also maintained in developmental leaf senescence (Fig. 6B). Expression levels of other genes related to protein synthesis and ribosome biogenesis were also maintained in *ahk3*/*ore12* (Fig. 5B) and BA-treated wild type (Supplementary Figs S3E, S4), but not in *ein2*/*ore3*. These data explain the fewer transcriptome changes in *ahk3*/*ore12* than in *ein2*/*ore3* (Fig. 3A), despite their comparable delayed senescence phenotypes (Fig. 1A–C; Supplementary Fig. S5). The transcriptional maintenance of RPs and related genes by cytokinin probably results in the accumulation of cytosolic, chloroplast, and mitochondrial RPs, which contribute to the self-maintenance activities of homeostatic proteins by counteracting degradation of cellular mRNA and proteins during leaf senescence (Diaz-Mendoza *et al.*, 2016).

Proteolysis is an essential process during senescence as it frees amino acids and peptides for use in developing seeds and other organs. The primary targets of proteolysis are chloroplast proteins including stromal proteins, Rubisco and GLUTAMINE SYNTHETASE 2, and thylakoid proteins, D1 and light harvesting complexes II (Prins *et al.*, 2008; Wada *et al.*, 2009; Simova-Stoilova *et al.*, 2010). Plants might have evolved cellular programs to maintain protein levels throughout cytokinin-mediated translational regulation of cytoplasmic mRNAs during senescence. The effect of cytokinin on protein accumulation has been reported in many plants, such as Norway spruce (*Picea abies*), soybean (*Glycine max*), tobacco, and Arabidopsis (Jouanneau, 1975; Fosket *et al.*, 1977; Maaß and Klämbt, 1977; Stabel *et al.*, 1990; Jain *et al.*, 2008); however, the mechanism has not yet been elucidated. Transcriptome profiling of transgenic tobacco plants expressing *pSARK::IPT* revealed up-regulation of nine *RP* genes caused by the high cytokinin levels (Rivero *et al.*, 2010). Thus, cytokinin-mediated protein accumulation throughout ribosome synthesis provides another regulatory layer for self-maintenance during leaf senescence.

The possible role of cytokinin in the inhibition of cellular protein degradation needs to be explored further. Also, it will be necessary to study in greater detail which proteins are selected for cytokinin-mediated translational regulation, how this selectivity is achieved, and what the mechanisms are by which cytokinin controls this process. Multi-omics approaches combining transcriptomics and proteomics could facilitate advances in these areas (Kim *et al.*, 2016). While many questions remain to be addressed, the present study advances our knowledge of the functions of ethylene and cytokinin in regulating leaf senescence and opens up new avenues of research in this interesting area of plant biology.

## Supplementary data

Supplementary data are available at *JXB* online.

Fig. S1. Transcription factor (TF) co-expression network in Arabidopsis *ein2*/*ore3* and *ahk3*/*ore12* mutants during dark-induced leaf senescence.

Fig. S2. Transcript profiles of genes belonging to gene ontology biological processes (GOBPs) significantly affected in *ein2*/*ore3* during dark-induced leaf senescence.

Fig. S3. Transcript profiles of genes belonging to GOBPs significantly affected in *ahk3*/*ore12* during dark-induced leaf senescence.

Fig. S4. Transcript profiles of genes encoding differentially localized ribosomal proteins (RPs) in Col, *ein2*/*ore3*, and *ahk3*/*ore12* treated with cytokinin in the dark.

Fig. S5. Phenotypes of Col, *ein2*/*ore3*, and *ahk3*/*ore12* leaves during developmental senescence.

Table S1. Primers used in this study.

Dataset S1. Genome-wide transcriptome analysis in Col, *ein2*/*ore3*, and *ahk3*/*ore12* during dark-induced leaf senescence

Supplementary Dataset

Supplementary Figure

## Abbreviations:

ACC: 1-aminocyclopropane-1-carboxylic acid
BA: 6-benzylaminopurine
Col: Columbia-0
DAE: days after leaf emergence
DAT: days after treatment
DEG: differentially expressed gene
GOBP: gene ontology biological process
GOCC: gene ontology cellular compartment
GOMF: gene ontology molecular function
JA: jasmonic acid
Pcc: Pearson’s correlation coefficient
PSII: Photosystem II
qPCR: quantitative real-time PCR
RP: ribosomal protein
SA: salicylic acid
TF: transcription factor.
