# Supplementary material for: Comparative transcriptome analysis in Arabidopsis ein2/ore3 and ahk3/ore12 mutants during dark-induced leaf senescence
Source: J Exp Bot. 2018 Apr 10;69(12):3023–36. doi: 10.1093/jxb/ery137 (PMC5972659; doi:10.1093/jxb/ery137)
Supplement: Supplementary Figure [file ery137_suppl_supplementary_figure_table_s1.pdf]

Journal of Experimental Botany

Comparative transcriptome analysis in Arabidopsis *ein2/ore3* and *ahk3/ore12* mutants during dark-induced leaf senescence

Jeongsik Kim[1,*], Su Jin Park[2,*], Il Hwan Lee[1,†], Hyosub Chu[1], Christopher A. Penfold[3], Jin Hee Kim[1], Vicky Buchanan-Wollaston[4], Hong Gil Nam[1,5], Hye Ryun Woo[5,‡], and Pyung Ok Lim[5,‡]

[1] Center for Plant Aging Research, Institute for Basic Science (IBS), Daegu 42988, Republic of Korea

[2] School of Interdisciplinary Bioscience and Bioengineering, POSTECH, Pohang, Gyeongbuk 37673, Republic of Korea

[3] Wellcome Trust/Cancer Research UK Gurdon Institute, University of Cambridge, Cambridge CB2 1QN, United Kingdom

[4] Warwick Systems Biology Centre, University of Warwick, Coventry CV4 7AL, United Kingdom

[5] Department of New Biology, DGIST, Daegu 42988, Republic of Korea

[*] These authors contributed equally to this work.

[†] Current address: Department of Forest Genetic Resources, National Institute of Forest Science, Suwon, 16631, South Korea

[‡] Corresponding authors:

Pyung Ok Lim

Phone: +82-53-785-1830

E-mail: polim@dgist.ac.kr

Hye Ryun Woo

Phone: +82-53-785-1870

E-mail: hrwoo@dgist.ac.kr

Supplementary materials and methods

Treatment of ethylene inhibitor

The third and fourth leaves of 3-week-old plants were detached and treated with 3 mM MES (pH5.7) buffer containing 5 μM silver nitrate (Sigma, USA) for 1 h. Leaves were washed with water briefly and floated on 3 mM MES (pH 5.7) for the indicated days.

Supplementary figures and tables

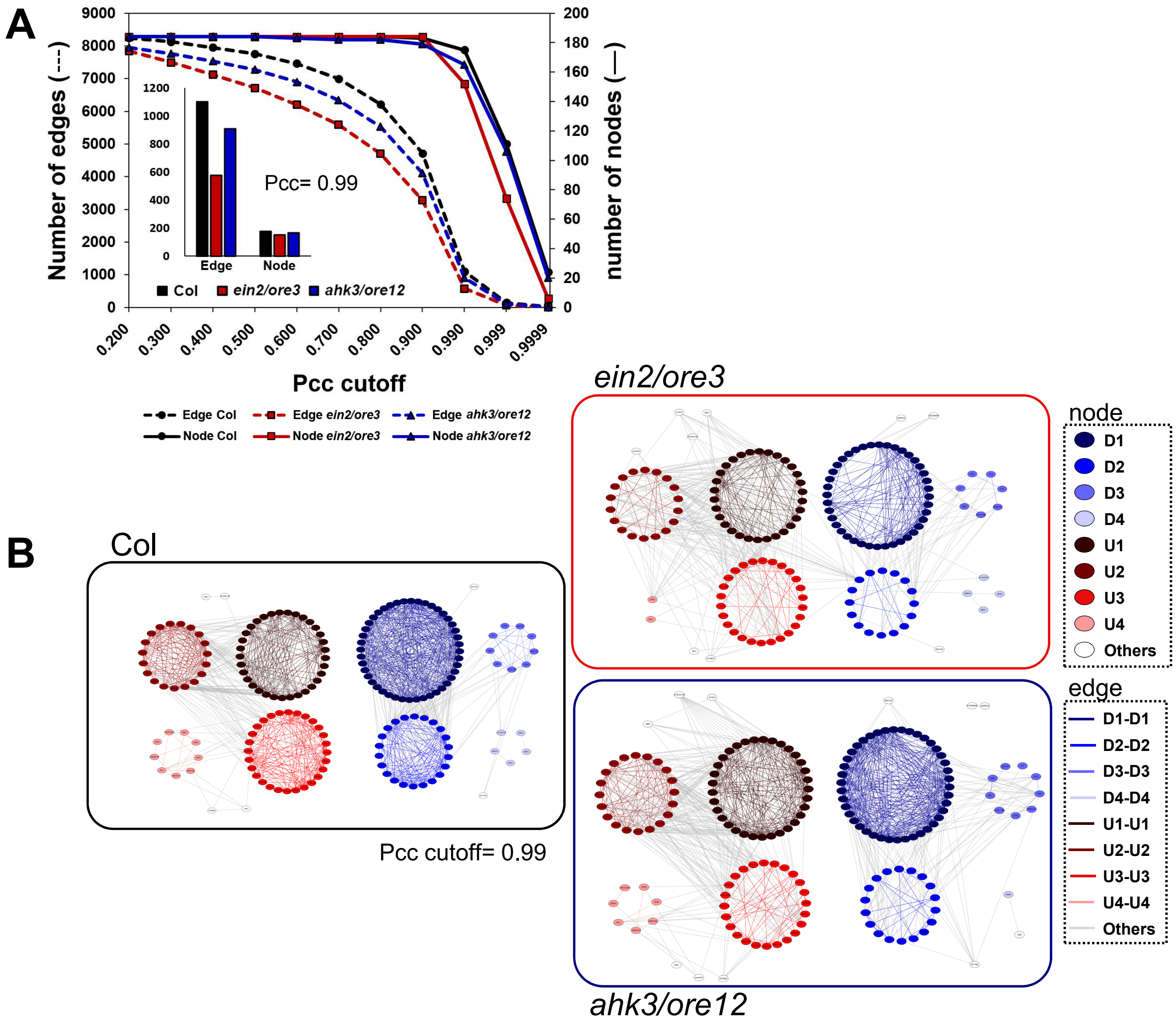

Fig. S1. Transcription factor (TF) co-expression network in Arabidopsis *ein2/ore3* and *ahk3/ore12* during dark-induced senescence. (A) The number of TF nodes and edges as a function of Pearson's correlation coefficient (Pcc) cut-off value in Col, *ein2/ore3*, and *ahk3/ore12*. Dashed lines indicated the number of edges and solid lines designate the number of nodes. Edges with Pcc larger than cut-off value indicated and nodes connected by these edges were included. TFs among the gene list described in Fig. 2A were used. Inset shows the number of TF nodes and edges at a Pcc cut-off of 0.99. (B) TF co-expression network in Col, *ein2/ore3*, and *ahk3/ore12*. The color of a node represents the cluster to which the TF belongs. Colored and grey edges indicate links between nodes belonging to the same and different clusters, respectively.

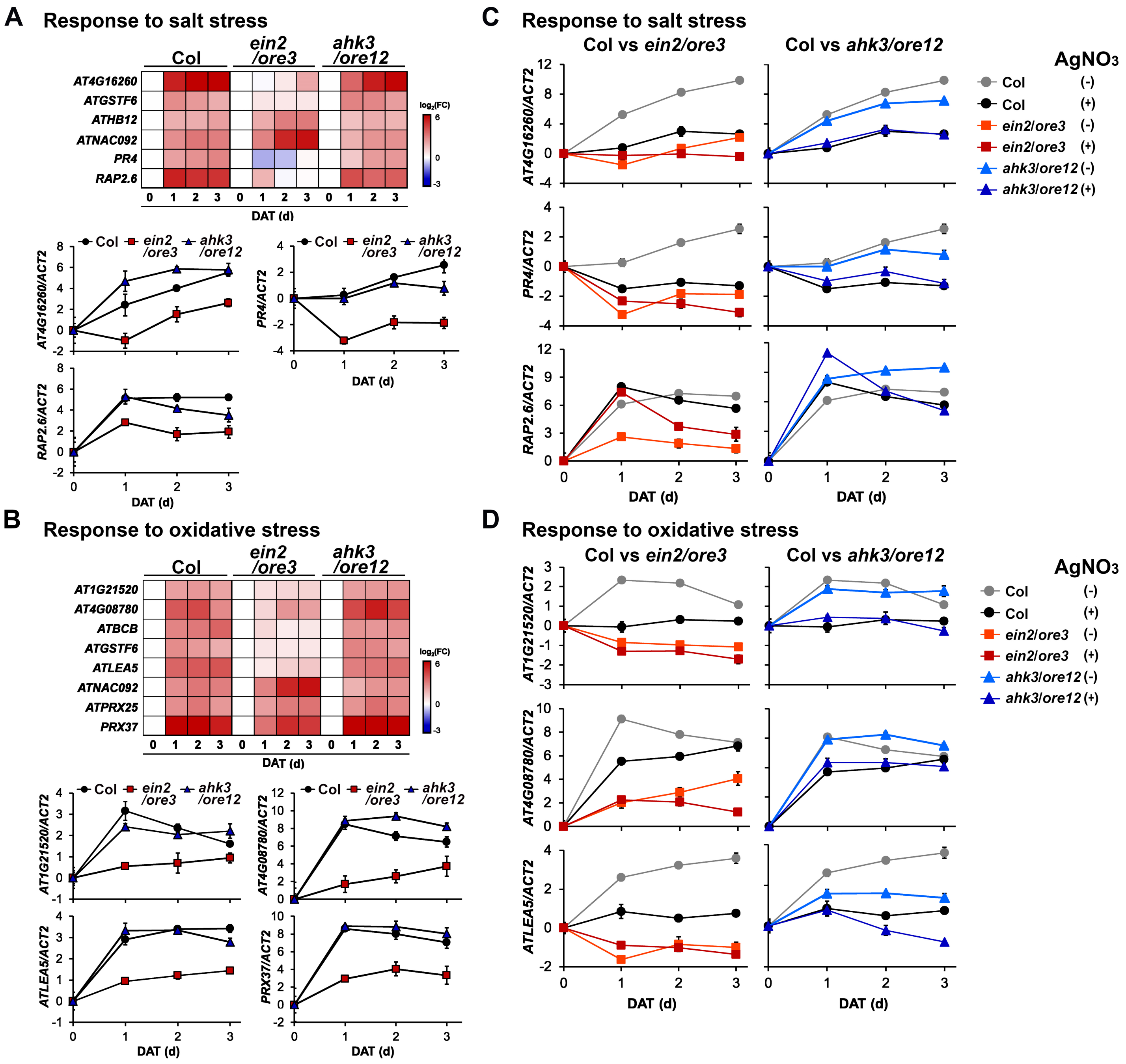

Fig. S2. Transcript profiles of genes belonging to gene ontology biological processes (GOBPs) significantly affected in *ein2*/*ore3* during dark-induced leaf senescence. (A, B) Differential kinetic expression of genes associated with the GOBP, responses to salt stress (*AT4G16260*, *PR4,* and *RAP2.6*) (A) and genes associated with the GOBP, responses to oxidative stress (*AT1G21520*, *AT4G08780, ATLEA5,* and *PRX37*) (B) from the analyses of microarray (upper panel) and qPCR (lower panel) data in Col, *ein2*/*ore3*, and *ahk3*/*ore12* at the number of days indicated. (C, D) qPCR results showing transcript levels of genes related to salt (C) and oxidative stress (D) response in Col, *ein2*/*ore3*, and *ahk3*/*ore12* leaves when treated with 5 μM $AgNO_3$ in the dark. Detached leaves were treated with or without $AgNO_3$ for 1 h, washed with water, and kept in the dark for 3 d. Kinetic patterns of gene expression are shown as $\log_2$ ratio of gene expression at the indicated time relative to that at day 0 in each genotype. Data represent mean ± SE (n = 3).

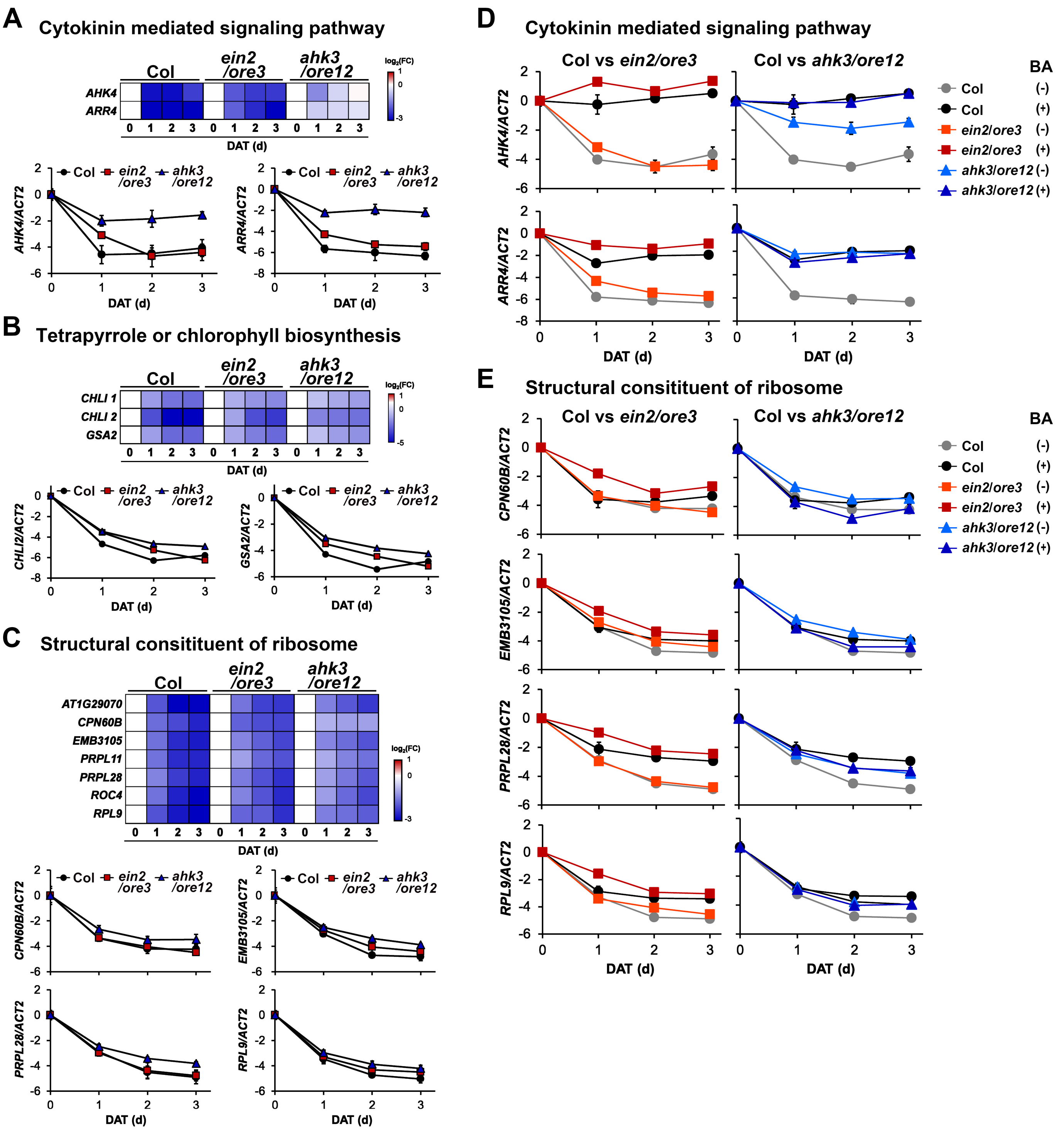

Fig. S3. Transcript profiles of genes belonging to GOBPs significantly affected in *ahk3/ore12* during dark-induced senescence. (A-C) Differential kinetic expression of genes associated with the GOBP, cytokinin-mediated signaling pathway (*AHK4* and *ARR4*) (A) and tetrapyrrole or chlorophyll biosynthesis (*CHLI2* and *GSA2*) (B), genes associated with GOMF, structural constituent of ribosome (*CPN60B, EMB3105, PRPL28,* and *RPL9*) (C), from the analyses of microarray (upper panel) and qPCR (lower panel) data of Col, *ein2/ore3*, and *ahk3/ore12* at the indicated days (DAT). (D, E) qPCR data showing transcript level of genes related to cytokinin signaling (D) or ribosome complex (E) in Col, *ein2/ore3,* and *ahk3/ore12* leaves treated with BA in the dark. BA treatment was performed as described in Fig. 1F, G. Kinetic patterns of gene expression are shown as the $\log_2$ ratio of gene expression at the indicated time relative to that at day 0 in each genotype. Data represent mean ± SE (n=3).

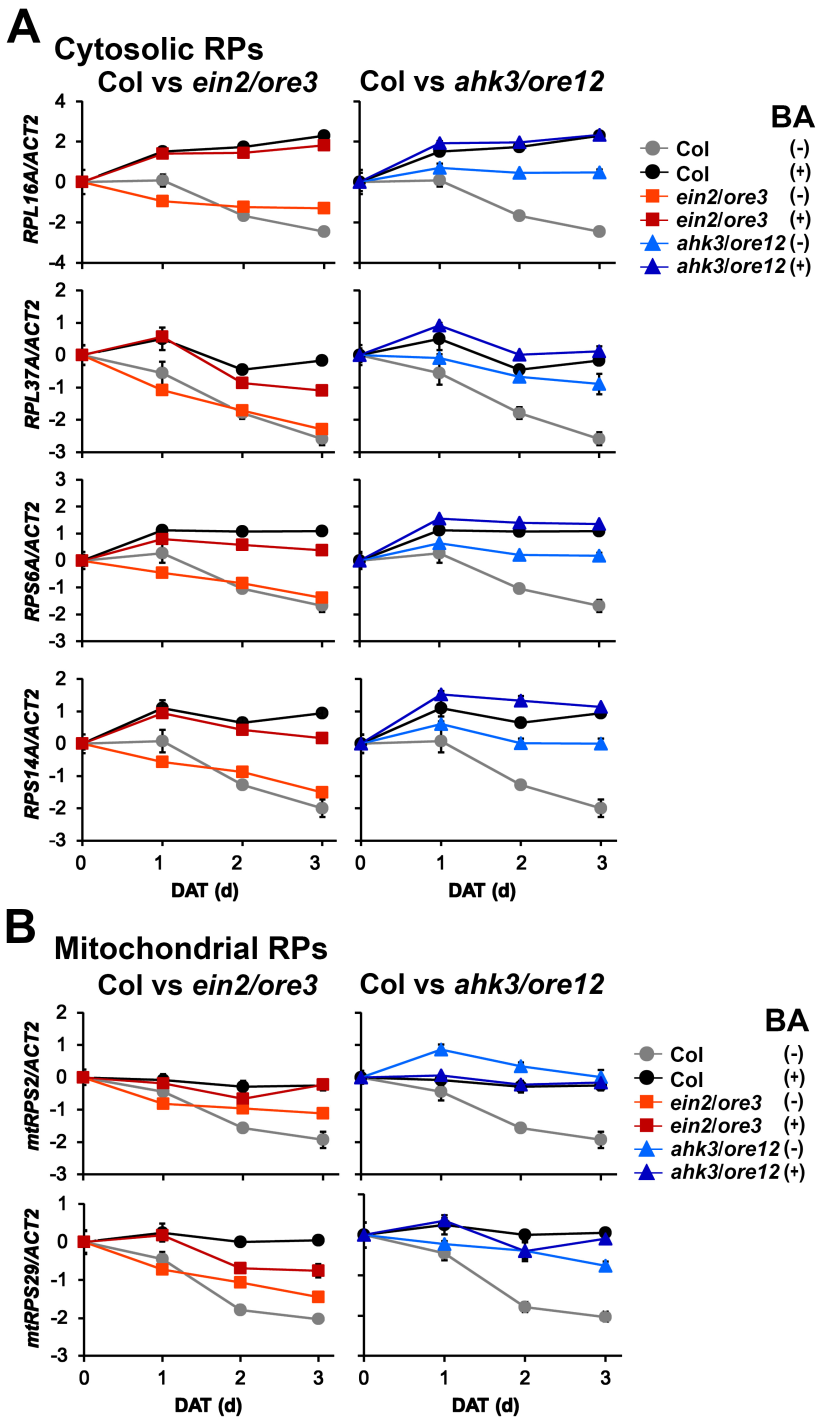

Fig. S4. Transcript profiles of genes encoding differentially localized ribosomal protein (RPs) in Col, *ein2/ore3*, and *ahk3/ore12* treated with cytokinin in the dark. (A, B) qPCR data showing transcript level of genes related to cytosolic (A) and mitochondrial (B) ribosome complex in Col, *ein2/ore3*, and *ahk3/ore12* leaves treated with BA in the dark. BA treatment was performed as described in Fig. 1F, G. Kinetic patterns of gene expression are shown as the $\log_2$ ratio of gene expression at the indicated time relative to that at day 0 in each genotype. Data represent mean ± SE (n=3).

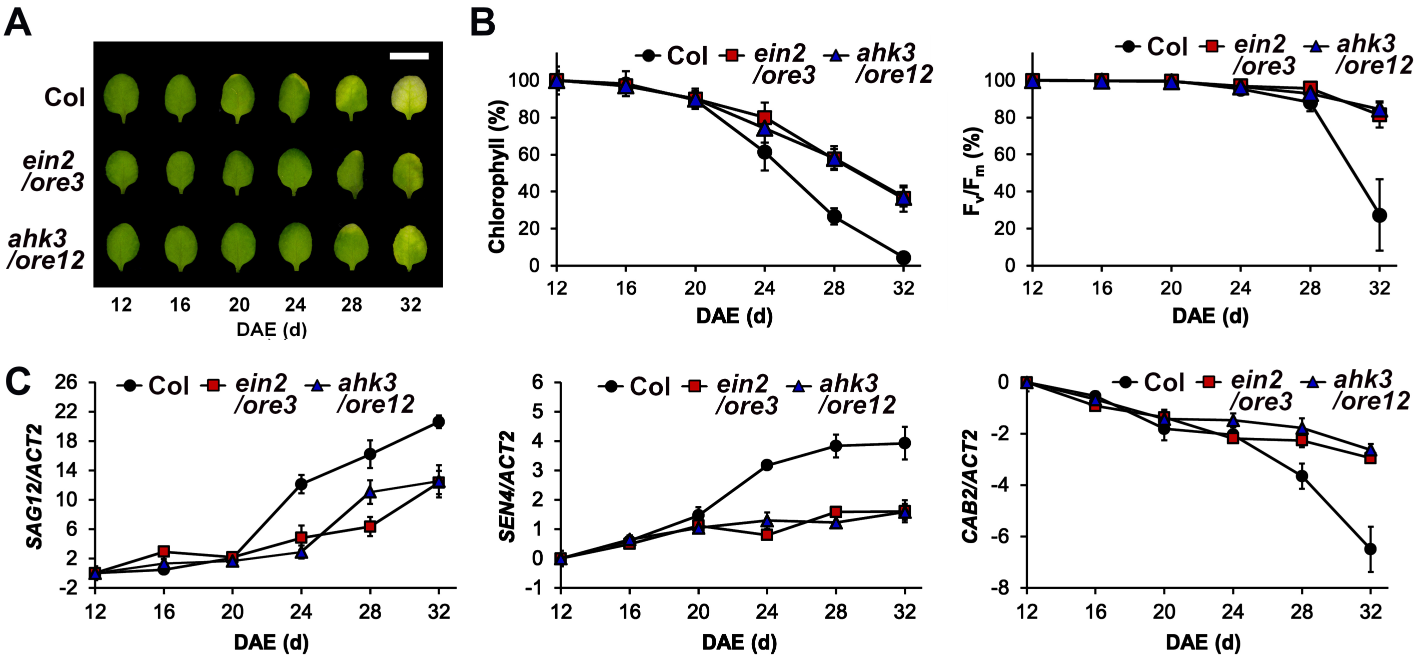

Fig. S5. Phenotypes of the Col, *ein2*/*ore3*, and *ahk3*/*ore12* leaves during developmental senescence. (A, B) Representative images (A), and measurements of chlorophyll content (B, left panel) and photochemical efficiency of PS II (F_v/F_m; B, right panel) of Col, *ein2*/*ore3*, and *ahk3*/*ore12* leaves of indicated ages (d; DAE). Scale bar = 1 cm. Data represent mean ± SE (n=12). (C) Changes in transcript levels of senescence-associated genes (*SAG12*, *SEN4*, and *CAB2*) in Col, *ein2*/*ore3*, and *ahk3*/*ore12* leaves of indicated ages. Kinetic patterns of gene expression are shown as the $\log_2$ ratio of gene expression at the indicated DAE relative to that at DAE 12 in each genotype. Data represent mean ± SE (n=4).

Table S1 Primers used in this study

Gene name	AGI	Purpose	Primer name	Sequence (5' → 3')
ACT2	AT3G18780	qRT-PCR	ACT2-F	CAGTGTCTGGATCGGAGGAT
			ACT2-R	TGAACAATCGATGGACCTGA
AT4G16260	AT4G16260	qRT-PCR	At4g16260-F	TGATATGACTTTGATTGGAAACTCTT
			At4g16260-R	AACGCTGAGTTCGTACTCGTAA
PR4	AT3G04720	qRT-PCR	PR4-F	TCCGACCAACAACTGTCAGA
			PR4-R	GTACGTTCGACGCGCTCT
RAP2.6	AT1G43160	qRT-PCR	RAP2.6-F	GGACGATGGGTCATAAGAGAGA
			RAP2.6-R	TGAGCTTTCACATTCTTTAGTCACA
AT1G21520	AT1G21520	qRT-PCR	At1g21520-F	CCTGCGACGGTAAACAGAG
			At1g21520-R	CAGTAGCGAGTGGGATACACG
AT4G08780	AT4G08780	qRT-PCR	AT4G08780-F	CAGCTCGAGGTTTCGATGTA
			AT4G08780-R	TGAAACGGTTCTAGGACATGC
ATLEA5	AT4G02380	qRT-PCR	ATLEA5-F	CATTCCTCGGATATATCTCTCCTT
			ATLEA5-R	TTCTTGAAAAGAGAGAAAACAGACTTT
PRX37	AT4G08770	qRT-PCR	PRX37-F	CGGCTCAAGAATCTGTCGTT
			PRX37-R	CCTTAAGTTGGTTAAGGGTAAAGAATG
AHK4	AT2G01830	qRT-PCR	AHK4-F	ACCGTTGCTAAGTGGAGTGGCTTA
			AHK4-R	ATATTCTCACGATCCTCCTCGCCT
ARR4	AT1G10470	qRT-PCR	ARR4-F	CTCTCGCGCCTCCACGTG
			ARR4-R	CGAAGGTTCGATTGCAATTTTCCC
CHLI2	AT5G45930	qRT-PCR	CHLI2-F	TCTTCAATGTCCCATCTCTGC
			CHLI2-R	TGTTCTTCTTTGGATTGAATTGG
GSA2	AT3G48730	qRT-PCR	GSA2-F	GCTTTCAATGCTGCGAAGA
			GSA2-R	CTTATTCGTGAGCCCTTTGC
CPN60B	AT1G55490	qRT-PCR	CPN60B-F	AGAGGTACACTGAAGATAGCAGCTC
			CPN60B-R	TTCCTCACGGATCACAGTTG
EMB3105	AT1G48350	qRT-PCR	EMB3105-F	CCTCTGGACCAACCATTGAG
			EMB3105-R	GTGATACCTTTCTCCAAGCAAGA
PRPL28	AT2G33450	qRT-PCR	PRPL28-F	CCGTATCTCCCTTCCTTCGT
			PRPL28-R	AACTTTGTTTGCTCTGTTTGCTT
RPL9	AT3G44890	qRT-PCR	RPL9-F	CCACTTCTGCTCAAGGAATTG
			RPL9-R	TACCATCGCCAGTTGTTGTG
RPL6A	AT1G33120	qRT-PCR	RPL6A-F	GTCTATCGAGATCCGTAAC
			RPL6A-R	CTCAGATCGAACAATGGTTAC
RPL6B	AT4G10450	qRT-PCR	RPL6B-F	GATTCGTAACTTCCTTGGTGAG
			RPL6B-R	CAAGCTCAATATCATTTCCCTC
RPL13	AT3G49010	qRT-PCR	RPL13-F	CCCTCGTCCAACTTCTGGACCT
			RPL13-R	ACGACGCGGGAAAATGACTAACTTG
RPL16A	AT2G42740	qRT-PCR	RPL16A-F	AAGTCAAGGAATACGAACTTCTCAG
			RPL16A-R	GATTCCAGTTGATGGATCGTACTT
RPL22e	AT3G05560	qRT-PCR	RPL22e-F	CACCTTCTAGGGTTTGTTCAAGA
			RPL22e-R	AGGAAACTCCCTTCTTCTTTCC
RPL28e	AT4G29410	qRT-PCR	RPL28e-F	TCAACTCGTACAAGCACTCTGG
			RPL28e-R	TGGTGGTTCCGAGTACAACA
RPL30/7	AT2G01250	qRT-PCR	RPL30/L7-F	GCTTAAGGGAGGTTTCTATG
			RPL30/L7-R	CTTAGGGTCAATGGCATTG
RPL37A	AT3G16080	qRT-PCR	RPL37A-F	GCACACTACTCGCTCCAATG
			RPL37A-R	ACGACGACCACATCTCACAC
RPL37B	AT1G15250	qRT-PCR	RPL37B-F	AGCTGCCAGAAAGAGGACATA
			RPL37B-R	CCTCATCCTACCAGTTCCTGTT
RPL40A	AT2G36170	qRT-PCR	RPL40A-F	TGTGGCCATAGCAACCAGT
			RPL40A-R	ACGTAAATAAGCTTTTGGTGAACA
RPS3C	AT5G35530	qRT-PCR	RPS3C-F	CGTTACAAGCTTCTTGGTGGT
			RPS3C-R	CTTCGCATCCTTTAGCTCCA
RPS4A	AT5G07090	qRT-PCR	RPS4A-F	GAGGCTCAATGCTCCTAAGC
			RPS4A-R	TGAGGTCCAGAAGATGGTTTG
RPS5	AT2G09990	qRT-PCR	RPS5-F	CAAGCGTGGATCTGGTTTAAT

			RPS5-R	GAGAAGAATCGGCTCGAAGA
RPS6A	AT5G10360	qRT-PCR	RPS6A-F	TACCGCCGTACCTTCACAA
			RPS6A-R	TCTGGAGGGTCAATGGAGTC
RPS13A	AT4G00100	qRT-PCR	RPS13A-F	GTCGTATGCACAGTCGAGGA
			RPS13A-R	CCTGAGAGGTGGTCTTGAGC
RPS14A	AT2G36160	qRT-PCR	RPS14A-F	TGAAAATAGGCCGTATTGAGG
			RPS14A-R	CCCACAATGCGAGAAAAACT
RPS16B	AT3G04230	qRT-PCR	RPS16B-F	AGTATCGCTAAGGCCCTCGT
			RPS16B-R	TCAAGATGTCCTTGATCTCCTTC
RPS17	AT3G18880	qRT-PCR	RPS17-F	CAATCGATATGTCAAGCGTAC
			RPS17-R	CTTGAAGGATCCAATTTCACTC
RPS19	AT5G09500	qRT-PCR	RPS19-F	CCCTTCCCGTATCCGTAGA
			RPS19-R	TGCTTCAATTTTCGCTTTCC
RPS25D	AT4G39200	qRT-PCR	RPS25D-F	TCTCACTGAAGCTCCCAAGTT
			RPS25D-R	GCTAGCGACCCATTGATCC
RPSAB	AT3G04770	qRT-PCR	RPSAB F	GATTCTCACCGACCCAAGAA
			RPSAB R	CCGTGTCACAGAAGGCAATA
PRPL4	AT1G07320	qRT-PCR	PRPL4-F	CGAGGTACGGTGTTGATGC
			PRPL4-R	CAATTGGAACTACGGGTGCT
PRPL11	AT1G32990	qRT-PCR	PRPL11-F	CCTCAACTCCGAGATTTCTCAC
			PRPL11-R	AAGCAAGTTTGATAACTCCCACA
PRPL12	AT3G27850	qRT-PCR	PRPL12-F	CGACTCTCTCAATCGCAACA
			PRPL12-R	GAGTGGAAGCGGAAGTGAGA
PRPL13	AT1G78630	qRT-PCR	PRPL13-F	GCAGCAGAGAATTCCTGAAAG
			PRPL13-R	CAGGGCCTTTGTACACCTTC
PRPL21	AT1G35680	qRT-PCR	PRPL21-F	TGCAAATGTTGATGACCAGATT
			PRPL21-R	ACCTTGACTTTCCACAACAGC
PRPL27	AT5G40950	qRT-PCR	PRPL27-F	CCAAGAACGGTCGTGATTC
			PRPL27-R	TTTTTCCCAGCATGGAACTT
PRPL34	AT1G29070	qRT-PCR	PRPL34-F	AATTGCTTCATTGCTCGTTTC
			PRPL34-R	GATCAAACGCAATGGACAAA
AT4G17560	AT4G17560	qRT-PCR	At4g17560-F	AGGCTTTACTATCTGAGGGACAAG
			At4g17560-R	ACGGTGGATGGTGTTCACTT
RFC3	AT3G17170	qRT-PCR	RFC3-F	GTGACTTGAATGAAGAAAGGATGA
			RFC3-R	GGTAATCTTGTACTTTCTGGTTAATGC
PSRP3/1	AT1G68590	qRT-PCR	PSRP3/1-F	ACACTCCACAACAAACAATAAAGC
			PSRP3/1-R	TGGTCCAATCCTAATCCTATGTTC
PSRP4	AT2G38140	qRT-PCR	PSRP4-F	TCCAGCTCAATTCCACTCGT
			PSRP4-R	CGCATTCCCAAAAGAGTGAT
PRPS20	AT3G15190	qRT-PCR	PRPS20-F	ATGCGTCGCCTTCTCAAC
			PRPS20-R	GCAGCCTCACACACAATCAA
PRPS21	AT3G27160	qRT-PCR	PRPS21-F	GCAGCAGCAACTAAGAGTAAGAAA
			PRPS21-R	TCAAGAAGGTACATCTCCACCAG
mtRPL16	ATMG00080	qRT-PCR	mtRPL16-F	CTTTCATATCGAGCCATTG
			mtRPL16-R	CTTACCATTTCTTCGGAATTG
mtRPS2	AT3G03600	qRT-PCR	mtRPS2-F	GAATGACAATTATCGACTCCGAC
			mtRPS2-R	GAAGATGTTACCTCTCATGTG
mtRPS7	AT2G07696	qRT-PCR	mtRPS7-F	AAGAGGGGAAGTGCACGTAG
			mtRPS7-R	TGTGGTCTCACTTTACCACCA
mtRPS29	AT1G16870	qRT-PCR	mtRPS29-F	TCCGTCTCTAGATGTTGGTCCT
			mtRPS29-R	GCTTTCAGTGCTGCCTCAT
mtRPS31	AT2G21290	qRT-PCR	mtRPS31-F	GCTTGAGCTTCCCAGGTCTA
			mtRPS31-R	AGGAGAAAACCCCTAACAAGAGA
